# Co‐designing a just‐in‐time adaptive mHealth intervention to improve parental support for child physical activity using a no‐code app design platform: Development study

**DOI:** 10.1111/aphw.70096

**Published:** 2025-11-29

**Authors:** Amanda Willms, Anna Sui, Rebecca Jantzen, Sean Chester, Leigh M. Vanderloo, Ryan E. Rhodes, Sam Liu

**Affiliations:** ^1^ School of Exercise Science, Physical and Health Education University of Victoria Victoria BC Canada; ^2^ Faculty of Engineering and Computer Science University of Victoria Victoria BC Canada; ^3^ Research & Evaluation, ParticipACTION Toronto Canada; ^4^ School of Occupational Therapy Western University London Canada

**Keywords:** co‐design, just‐in‐time adaptive intervention, mobile health, physical activity

## Abstract

Parental support significantly influences children's physical activity (PA) levels. Just‐in‐time adaptive interventions (JITAIs) delivered through mobile health (mHealth) may provide personalized, dynamic support to parents, though research is limited. 1) Describe the co‐design process of a family‐based JITAI app designed to help parents support their children's PA, and 2) evaluate the resources required to co‐design this app using a “no‐code” platform, Pathverse. Following the Integrate, Design, Assess, Share (IDEAS) and Multi‐Process Action Control (M‐PAC) frameworks, parents of children 8–12 years not meeting PA guidelines participated in semi‐structured interviews (Phase 1). Feedback‐informed app features, JITAI tailoring strategies, and prototype refinement (Phase 2). Six parents participated in Phase 1 guided by the IDEAS framework, with parental feedback directly shaping the app design. Parents emphasized family‐based content, gamification, and diverse PA activities, while barriers (e.g., time, weather) informed JITAI tailoring. The M‐PAC framework guided the selection and delivery of behavior change techniques (e.g., self‐monitoring, social support). Development required 320 hours over four months, including decision‐tree creation (50), uploading dynamic content (70), and testing (80). A family‐based JITAI app was co‐designed leveraging the M‐PAC framework and Pathverse to integrate parental support for PA, laying the groundwork for future testing.

## BACKGROUND

Physical inactivity is an important modifiable risk factor for reducing chronic disease, including cardiovascular disease, cancer, diabetes, mental health, and premature mortality (Dhuli et al., 2022). While chronic diseases predominantly affect older adults, their development often begins earlier in life, underscoring the importance of initiating preventive measures during childhood (Warburton & Bredin, 2017). By fostering healthy behavioral patterns early on, these interventions can have long‐lasting effects, helping to mitigate the onset of chronic diseases later in life (Dalal et al., 2022). Further, achieving adequate physical activity (PA) in childhood has a range of benefits beyond chronic disease prevention, including improving bone health (Janz et al., 2010), social skills (Erdilanita & Ma'mun, 2024), family functioning (Rhodes et al., 2024), and cognitive health and performance (Biddle et al., 2019). Family‐based interventions that support parents in encouraging their children's PA have been shown to be effective in increasing child PA (Huang et al., 2011; Liu et al., 2020; Perdew, Liu, & Naylor, 2021; Rhodes, Guerrero, et al., 2020). However, challenges remain in scaling and personalizing these interventions, as parents often face barriers such as limited time, competing responsibilities, and lack of access to resources (Kilfoy et al., 2025).

To address these challenges, emerging strategies leveraging the Internet and mobile technology have shown promise (Liu, Smith, et al., 2022; Perdew, Liu, Rhodes, et al., 2021; Willms et al., 2023a). In particular, just‐in‐time adaptive interventions (JITAIs) delivered through mobile applications (apps) can use real‐time data and algorithms to provide personalized support to parents in promoting their children's PA (Hardeman et al., 2019; Wang & Miller, 2020). Personalization in these interventions allows for tailored support that can adapt to the unique needs, preferences, and circumstances of each individual, increasing the likelihood of engagement and success. JITAIs offer several advantages over traditional non‐adaptive mobile health (mHealth) PA interventions. Specifically, JITAIs can use PA data (e.g., self‐report or wearable measures) to deliver behavioral support that aligns directly with the user's needs (e.g., prompting PA suggestions matched to parents' motivation). Furthermore, JITAIs can dynamically tailor the content and timing of behavioral support based on input data across various timescales and contexts. This means the interventions can adjust according to the user's current motivation and confidence levels regarding PA over time (Müller et al., 2017). Although JITAIs have been used successfully in individual‐focused PA interventions, extending their application to family‐based approaches presents unique opportunities to address barriers and promote sustainable PA behaviors within households. Family‐based JITAIs could leverage real‐time data to support not only individual behavior change but also collaborative family dynamics that influence children's PA.

There is currently a lack of family‐based JITAIs aimed at supporting parents in promoting children's PA due to the complexity of designing intervention‐tailoring algorithms that can effectively adapt to parental support behaviors in real time, as well as the high costs and resource demands involved in co‐designing these interventions. However, these challenges can be mitigated through strategic and collaborative design approaches. For instance, co‐designing mHealth apps with end users is crucial for creating interventions that are user‐centered, acceptable, and effective (Saparamadu et al., 2021). Involving users in the development process ensures that the app meets their needs, accounts for their preferences, and addresses potential barriers to usage (Noorbergen et al., 2021). This collaborative approach enhances user engagement and increases the likelihood of sustained intervention success. Further, incorporating co‐design practices aligns well with the IDEAS (Integrate, Design, Assess, and Share) framework for mHealth development, which emphasizes iterative development and stakeholder involvement at every stage (Mummah et al., 2016).

Behavior change theories provide a critical foundation for designing effective JITAI tailoring algorithms (Cotie et al., 2025; Rhodes et al., 2024). These frameworks identify key psychological determinants of behavior change, their underlying behavior change techniques (BCTs), and guide the delivery of timely motivational support and practical strategies to encourage desired health behaviors. Although there are many theories of behavior change that have been applied to family‐based interventions (e.g., Social Learning Theory, Social Cognitive Theory, Self‐Determination Theory (Rhodes et al., 2024)), the Multi‐Process Action Control (M‐PAC) framework was used in this study to build upon these traditional approaches. The M‐PAC framework emphasizes how reflective, regulatory, and reflexive processes support the translation of intentions into sustained action (Kaushal et al., 2017; Rhodes, 2017; Rhodes & Yao, 2015; Vallerand et al., 2017). Reflective processes involve conscious evaluations, such as recognizing the benefits of supporting children's PA (instrumental attitude) and believing in one's capability to do so despite challenges (perceived capability). Regulatory processes help individuals prioritize and sustain behaviors through strategies such as goal setting, self‐monitoring, and emotion regulation. Reflexive processes are critical for long‐term maintenance, including habit formation and the development of behavior‐related identity, such as viewing oneself as a supportive and active parent. Previous research has applied M‐PAC in the domain of family support (Rhodes et al., 2016; Rhodes et al., 2019), making it particularly relevant to our focus on parental support for children's PA. Parents play a central role in shaping children's activity through modeling, encouragement, and creating supportive home environments (Rhodes, Guerrero, et al., 2020). Incorporating these family dynamics into JITAIs allows interventions to respond to real‐life contexts while fostering lasting behavior change.

Additionally, a “no‐code” mHealth development platform such as Pathverse can significantly reduce the resource demands of developing mobile apps and JITAIs (Liu, La, et al., 2022; Willms et al., 2023b). Pathverse allows researchers to build mHealth interventions without requiring coding expertise, potentially lowering the costs and resources associated with co‐designing these interventions compared to traditional software development methods. By utilizing “no‐code” platforms with the IDEAS framework, researchers can efficiently prototype, test, and refine JITAIs with direct input from end‐users. Thus, the primary objective of this study was to describe the co‐design process of a family‐based JITAI app, Movement for Active Families, designed for parents to support their child's PA. The secondary objective of this study was to evaluate the resources required to co‐design the JITAI app using Pathverse.

## METHODS

### Design

We followed the IDEAS framework (Mummah et al., 2016) to co‐design a family‐based JITAI PA promotion app called Movement for Active Families. This study used the first two phases of the IDEAS Framework (i.e., Integrate, Design) to iteratively plan and develop the JITAI app. The latter stages of the IDEAS framework (i.e., Assess, Share), which are relevant for intervention evaluation and dissemination, will be applied in future studies. We divided the study process into two relevant phases: 1) a co‐design and intervention planning phase, and 2) an intervention development phase. A summary of the development timeline is presented in Table 1.

**TABLE 1:** The planning and development phases of the JITAI.

Phase	Steps	Activities	Dates
1. Co‐design and intervention planning	1. Empathize with target users	Conducted semi‐structured interviews with parents of children 8–12 years to gather app requirements	August–October 2022
	2. Specify target behavior	Explored the feasibility of targeting child physical activity levels, but shifted to parental support for physical activity after researching privacy concerns	October–November 2022
	3. Ground in behavioral theory	Integrated content within the multi‐process action control framework	October–November 2022
2. Intervention development	4. Ideate implementation strategies	Used an iterative process to ideate, implementing input from participants and behavioral theory	December 2022–January 2023
	5. Prototype product	Used the Pathverse platform to rapidly develop a functional app prototype	February–March 2023

### Identifying user requirements

Target users were involved in the first phase of the study, and a multidisciplinary team was involved in phases 1 and 2 and recruited through convenience sampling methods. Target users were parents and/or primary caregivers (18 + years) of children 8–12 years who self‐reported that their child was not meeting the Canadian PA guidelines of 60 minutes of moderate‐to‐vigorous PA, that they were free of visual impairments, and were able to speak English during eligibility screening. This age group of children was of particular interest to us as they are more flexible than adults in their ability to change behaviors, as they are just beginning to develop self‐regulation skills, habits, and identities for healthy living (Pandita et al., 2016). Recruitment of target users continued until thematic saturation was reached, meaning no new themes or insights would be identified during the semi‐structured interviews. The multidisciplinary team consisted of domain experts in family‐based PA interventions (i.e., PA guidelines, activity modes, and program design; n = 2), behavior science (i.e., behavior change techniques, exercise psychology; n = 2), and app development (i.e., user interface design, user experience, app development; n = 2). Each phase lasted approximately four months in length and is detailed below.

#### Ethics approval

Ethics approval for this study was obtained from the Human Research Ethics Board at the University of Victoria (protocol number 22‐107). All participants provided written informed consent, and each participant was given a unique identifier to maintain anonymity for data analysis.

### Phase 1: co‐design and intervention planning (August–November 2022)

#### Step 1: empathize with target users

Two members of the research team (AW, AS) recruited parents (18 + years) from Facebook ads with children 8–12 years who were not currently meeting the Canadian PA guidelines of 60 minutes of moderate‐to‐vigorous PA per day. Participants were invited for a 60‐minute semi‐structured interview via video call. The purpose of these interviews was to learn about the family's current PA tendencies, to discuss potential features of the app, and to receive feedback on potential app content. Semi‐structured interview questions were kept generic at this phase to incorporate all possible answers and to avoid bias toward any particular theoretical framework. Interviews were recorded, and the researchers took field notes throughout the call. The research team has previously developed family‐based healthy lifestyle content (Liu, Marques, et al., 2019; Perdew, Liu, Rhodes, et al., 2021) and sought to determine whether the content was acceptable for this demographic. The questions used in this interview are presented in Table 2.

**TABLE 2:** Questions and probes from the semi‐structured interview.

Questions	Follow‐up questions
What kind of physical activity does your family currently engage in?	How do you motivate one another to engage in physical activity? What barriers do you encounter in participating in physical activity?
What kind of fitness/exercise app do you currently use to engage in physical activity, if any?	What are some of the features that you enjoy about this app? What are some features you feel are missing from this app? If you are not using an app, how come?
Imagine an adaptive physical activity intervention app:	What kind of content would you like to see? For example, this can include physical activity guidelines, ideas for physical activity, and nutrition information. Are there, if any, features that you would not like to see in this app?
Is there anything that we missed during this focus group that you would like to share?	*Probe*: Can you tell me more about that?

Sessions were audio‐recorded and transcribed verbatim using Transcriptive (Digital Anarchy, 2021) to ensure the accuracy of the data. After the semi‐structured interviews concluded, the research team (AW, AS) reviewed their field notes and then met to debrief and discuss broad concepts that emerged from the data. The transcripts were then input into Taguette (Rampin & Rampin, 2021) to be coded inductively. Thematic analysis (Braun & Clarke, 2006) was used to determine relevant themes and theoretical frameworks. From the debrief, broad semantic codes arose from the data that captured the essence of the semi‐structured interviews. These codes were the initial themes and were input into Taguette, with further themes emerging from analysis. Upon completing the thematic analysis, the team members (AW, AS) shared the results with the multidisciplinary team to proceed with the rest of phase 1.

#### Step 2: specify target behavior

Our target behavior was the improvement of children's PA levels. Our team met to identify specific metrics for measuring child PA, such as moderate‐to‐vigorous PA and steps, and to explore potential methods (wearable versus self‐report) for tracking these metrics in the Movement for Active Families JITAI.

#### Step 3: ground in behavioral theory

We used the M‐PAC framework to guide the JITAI development (Rhodes & Kwan, 2025). Adaptive smartphone‐based approaches are beneficial for the M‐PAC constructs as they can quickly address failed intentions by tailoring options to an individual's specific circumstance. These options can be delivered through behavior change techniques that align with M‐PAC constructs, such as plans, goals, self‐monitoring (regulatory processes), prompts and cues (needed for habit formation), adaptive responses to inaction (i.e., feedback from wearables and pre‐set goals) (Kaushal et al., 2017; Rhodes, 2017; Rhodes & Yao, 2015; Vallerand et al., 2017).

### Phase 2: intervention development (December 2022–March 2023)

#### Step 4: ideate implementation strategies

Our team co‐created app content, JITAI delivery logic, app user interface, and engagement features (e.g., rewards, forum). Our team created intervention content based on the literature and our previous M‐PAC trials (Liu, Marques, et al., 2019; Rhodes, Perdew, & Malli, 2020). We also used ChatGPT to help adjust the tone and style of the content to appeal to parents of children 8–12 years (Willms & Liu, 2024). The information gathered from phase one was synthesized and implemented into this 10‐week intervention. Specific to JITAI logic, we determined the decision points and decision rules (including tailoring variables, values of tailoring variables, and intervention options) (Nahum‐Shani et al., 2018).

#### Step 5: prototype potential products

We developed the Movement for Active Families app using the Pathverse platform (Pathverse Inc., 2022) over a four‐month period from December 2022 to March 2023. While prototyping potential products, the research assistant logged their hours to determine the amount of time spent on each task. Tasks involved content upload, exploring potential app color schemes, and iterative internal testing for usability and technical errors. Thorough documentation and regular collaboration between the multidisciplinary teams ensured that new design features aligned with semi‐structured interview findings, theory, empirical evidence, and capabilities of the Pathverse platform. The modifications to content, design, and features were documented and completed by the multidisciplinary team to ensure the accuracy and completeness of modifications.

## RESULTS

### Phase 1: intervention planning (August–November 2022)

#### Step 1: empathize with target users

Six parents (n = 5 mothers, n = 1 father) were recruited to share their experiences with using mobile apps for PA. No new themes emerged after our first four semi‐structured interviews, indicating our sample size was sufficient for saturation (Guest et al., 2020; Staller, 2021). The mean age of parents was 45.3 ± 2.6 years (range: 43–48), and the mean income was CAD $53,333.00 ± $41,413.94 (range: $30,000–$99,999). Parents came from a range of cultures, consisting of Indigenous Canadian (n = 1, 17%), Latin American (n = 1, 17%), South Asian (n = 2, 33%), and White (n = 2, 33%). Three parents were located in British Columbia (50%), and three parents were located in Ontario (50%). Their answers were coded into two overarching themes: app preferences and PA preferences. App preferences encompassed several subthemes relating to features and functionalities that the app is capable of, as well as the type of content delivered through the app. Specifically, most of the participants (n = 5) mentioned having a variety of PA options delivered through various multimedia formats (i.e., videos, photos, text instructions). They emphasized the importance of having a diverse range of activities to cater to different interests and fitness levels, including activities suitable for families to do together (n = 3). Further, most participants (n = 5) expressed a strong desire for some sort of reward system (i.e., gamification elements) that they can see within the app, which would serve as motivation and encouragement for consistent engagement. Additionally, participants stressed the need for the app to be user‐friendly (n = 3), with intuitive navigation and clear instructions to ensure that they could easily access and utilize the app to its full potential.

The second overarching theme, PA preferences, encompassed subthemes such as current PA tendencies, facilitators for PA, and barriers for PA. Participants shared a variety of activities they enjoyed doing together, including walks, hikes, bike rides, water activities, and sports at local facilities. These activities were not only seen as opportunities for PA but also as bonding experiences for families. The feedback indicated that the app should incorporate content and features that promote these specific activities and provide guidance on how to engage in them safely and effectively. Additionally, the participants highlighted various facilitators for PA, such as the importance of scheduling and routine in maintaining a healthy lifestyle (n = 2), and they emphasized the significance of having access to outdoor spaces and community resources (n = 2). On the other hand, participants also revealed several barriers to PA, including time constraints due to work and family commitments (n = 3), the lack of nearby recreational facilities (n = 1), and weather‐related challenges (n = 2). These insights suggest that the app should address these barriers by offering flexible workout schedules, indoor exercise options for adverse weather conditions, and guidance on making the most of limited time. During the coding process, the research team identified that the subthemes and user preferences corresponded closely with the constructs of the M‐PAC framework. As such, we mapped the subthemes and associated quotes to the relevant M‐PAC constructs and then stated the app feature (content or component) that each subtheme translated to. The results of this mapping are presented in Table 3.

**TABLE 3:** Subthemes and quotes provided relating to each subtheme and the associated M‐PAC construct, BCTs, and the app features they correspond to.

Subthemes	Quotes	M‐PAC construct; BCTs	App feature
App features liked	“They're [redeemable] points … You can spend [the points] on like to get dollars off basically a purchase like you, the points are like, so like, like 2 cents or something, but it like it racks up and it's just like, I don't know, just seeing the points there.” Participant 1 “[in the app I currently use] there's [feature that suggests] a goal where I can achieve that. It's not a [unrealistic] goal … It gives you a badge once you complete a certain accomplishment. Like if you're running 40kilometers, once you achieve it, they give you a badge.” Participant 3	Reflective processes; material incentive (10.1)* Regulatory processes; feedback on behavior (2.2)*, rewards (10.9)*	Points for finishing modules and family challenges that are redeemable for prize Families could mark family challenges as “done” and see log of completed challenges
Content preferences within app	“I'd love to see variety, like articles plus physical, like little challenges and maybe like wellness stuff, too. And then maybe like have different levels. Like if you're a beginner, like don't start out with like a [6 km run] like start out small and then yeah.” Participant 1 “[Educational content] would be interesting. Like can be something related to your goals.” Participant 4	Reflective processes; Graded tasks (8.7)* Reflective processes; information about health consequences (5.1)*	Provided text and video content, links to resources from reputable sources (i.e., PartipACTION, CSEP, and WHO) Received tailored content based on the M‐PAC construct parents indicated they needed support in
Current physical activity tendencies	“Mostly like walks and hikes … We've done geocaching. Or in the summertime we sometimes go canoeing and different activities like swimming.” Participant 1 “Mostly it is walking … In the summer, we go cycling sometimes” Participant 6	Reflexive processes; habit formation (8.3)*, identity associated with behavior (13.1)* Reflexive processes; behavioral practice/repetition (8.1)*	Highlighted mentioned activities, and similar, in family challenges (i.e., orienteering, links to trails nearby)
Facilitators for physical activity	“We just like doing stuff together. It keeps us healthy and it's kind of like a bonding time.” Participant 1 “I would like the idea of little games [we can do together] […] So I participate in it instead of just being like a cheerleader, you know what I mean? So, we're doing it together. Cause then we can motivate each other.” Participant 2 “For the kids, if we want to do a longer walk or a hike, usually there's like a desirable destination, like an ice cream shop or something going for something to eat or drink, or having friends along is also very motivating for them.” Participant 5	Regulatory processes; social support (3.1)* Regulatory processes; social support (3.1–3.3)* Reflective processes; Incentive reward (10.9)*, social reward (10.4)*	Family challenges and content focused on family self‐care and budget‐friendly family activities
Barriers for physical activity	“I think stress [at work or within the family is] one thing and workload, plus I think the weather. Weather is a big part. So, if the weather is not good, obviously you don't feel like going out.” Participant 3 “[The] biggest barrier is motivation, [the] lack of motivation. That's the hardest part to get [physical activity] in. I think if we have [motivation], the rest is actually done … If we have the motivation, we'll be doing it like every day … You deal with other big areas in your life, you know, finances, a job or disease. [This] takes a lot of time and energy from you, you know, that is stressors in life … Sometimes you don't have energy to put into like, you know, physical activity.” Participant 6	Reflective processes; problem solving (1.2)* Reflective processes; self‐motivation (15.1)*, emotional regulation (11.2)*	Content focused on overcoming common barriers to PA and tailored weekly content to support PA intention formation and adoption

*Note*: M‐PAC = Multi‐Process Action Control; BCT = behavior change technique; PA = physical activity; CSEP = Canadian Society for Exercise Physiology; WHO = World Health Organization; *The numbers in brackets indicate the behavior change technique for reporting in behavior change interventions (Michie et al., 2013).

#### Step 2: specify target behavior

**FIGURE 1:**
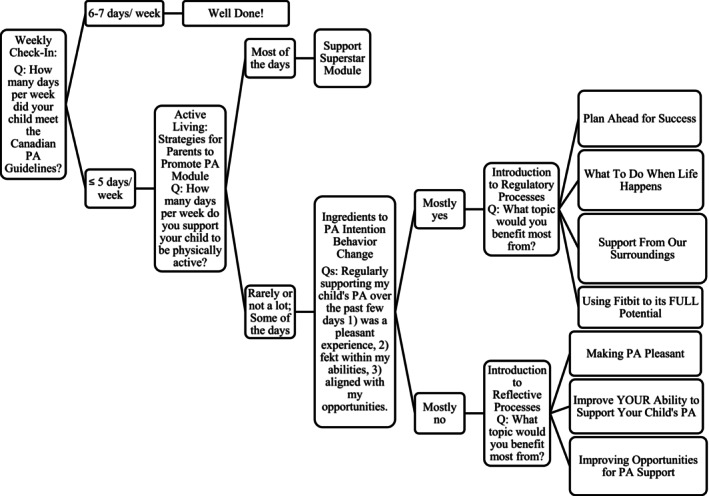
Decision tree to display just‐in‐time adaptive intervention decision rules. *Note*. At the highest level, the questionnaire asked how many days per week the child met the Canadian PA guidelines within the past 7 days. The options were 6–7 days per week, prompting a congratulations module, or 5 days or less per week, prompting an additional support module and follow‐up questions. The additional module detailed parental support for PA strategies, along with a question asking how many days they intentionally supported their child to engage in physical activity, with the choices 1) rarely or not a lot, 2) some of the days, and 3) most of the days. If the parent selected option 1 or 2, a subsequent question followed to determine whether they needed reflective support (either through an affective attitude module (“Making Physical Activity Pleasant”, a perceived capability module (“Improve YOUR Ability to Support Your Child's Physical Activity”), or an opportunities module (“Improving Opportunities for Physical Activity Support”)) or regulatory support (either through a proactive behaviors module (“Plan Ahead for Success”), a self‐monitoring‐focused module (“Using Fitbit to its FULL Potential”), a reactive regulatory module (“What to do When Life Happens”), or a restructuring one's social norm module (“Support From Our Surroundings”)).

Our team decided to focus the JITAI target behavior on children's PA levels, specifically, the number of days during the past week the child met the Canadian PA guidelines of 60 minutes of MVPA. We planned to capture child PA using Fitbit; however, based on Fitbit's privacy policy regarding accessing child PA data (Fitbit Privacy Policy for Children's Accounts, 2024), the target behavior was refined to parental self‐report measures of child PA. Due to the nature of this tailoring variable, real‐time, device‐driven feedback was not possible. Instead, parents were prompted to complete a brief questionnaire each Thursday (Figure 1). This input was our tailoring variable: once parents submitted their responses, they received tailored feedback and support content immediately. The decision to schedule tailoring late in the week was intentional, as it allowed parents to reflect on their child's recent PA patterns while still providing just‐in‐time support that could influence their weekend behaviors and carry momentum into the following week. While not real‐time in the sense of passive monitoring, the system provided context‐sensitive support based on parents' immediate reports, aligning with the “just‐in‐time” principle by ensuring that feedback was delivered at a critical transition point for behavior planning and change.

#### Step 3: ground in behavioral theory

In order to help improve child PA, the Movement for Active Families app focused on parental support for child PA. Our team developed 25 unique modules grounded in the M‐PAC framework that were delivered throughout the 10‐week intervention. Twenty modules, two modules per week, were released to all participants and began with intention formation, action control adoption, and action control maintenance. Additionally, we generated adaptive decision tree algorithms aimed at improving parental support for children's PA using the M‐PAC framework. The decision tree depicted in Figure 1 integrates reflective, regulatory, and reflexive processes from the M‐PAC model into the adaptive structure. The intervention begins by assessing the child's adherence to Canadian PA guidelines and follows up with tailored modules based on parental involvement in supporting their child's PA. For parents reporting lower support levels, the framework first determines whether their need lies in the reflective domain (e.g., attitude, perceived capability, or opportunity), directing them to modules like “Making Physical Activity Pleasant,” “Improve YOUR Ability to Support Your Child's Physical Activity,” or “Improving Opportunities for Physical Activity Support.” Alternatively, if the regulatory process is identified as a barrier, parents are routed to strategies that help enhance self‐regulation, including proactive behavior plans (“Plan Ahead for Success”), self‐monitoring (“Using Fitbit to its FULL Potential”), reactive strategies (“What to Do When Life Happens”), or social support restructuring (“Support from Our Surroundings”). This approach personalizes the intervention by addressing specific gaps in the parents' reflective or regulatory capacities, providing timely and targeted support to improve their child's PA. See Table 4 for module titles of both the traditional modules and the JITAI modules, objectives, M‐PAC constructs, and behavior change techniques used.

**TABLE 4:** Outline of weekly intervention content and JITAI modules, M‐PAC constructs used, and BCTs.

Week	Module name	Module objectives	M‐PAC construct(s)	BCTs
1	App tutorial and program foundation Physical activity guidelines	App tutorial Overview of program Introduce M‐PAC framework Introduce JITAI component Introduce family goal setting 24‐hour movement guidelines Benefits of PA	Reflective and regulatory processes	Action planning (1.4)*, information about health consequences (5.1)*
2	Parenting practices that promote health 23 and a ½ hours!	Strategies and opportunities for parents to better incorporate healthy living into daily family life Benefits of PA for chronic disease prevention	Reflective processes	Goal setting (1.1)*, action planning (1.4)*, self‐monitoring (2.3)*, behavioral practice/rehearsal (8.1)*, graded tasks (8.7)*
3	Increasing self‐confidence Setting yourself up for success	Introduction to self‐efficacy FITT principle Progression principle Setting SMART goals	Reflective and regulatory processes	Goal setting (1.1)*, self‐monitoring (2.3)*, behavioral practice/rehearsal (8.1)*, graded tasks (8.7)*, instruction on how to perform a behavior (2.1)*, information about health consequences (5.1)*, action planning (1.4)*
4	Breaking through barriers Fun for the whole family	Introduce affect Emotional regulation for PA Overcoming three common barriers to PA Social support	Reflective and regulatory processes	Goal setting (1.1)*, self‐monitoring (2.3)*, behavioral practice/rehearsal (8.1)*, graded tasks (8.7)*, instruction on how to perform a behavior (2.1)*, instruction on how to perform a behavior (2.1)*, social support (3.1–3.3)*
5	Building physical activity opportunity A new meaning to “home gym”	Built environment The social ecological model The influence of environment on behavior, focus on indoor Changing one's environment to promote PA, focus on indoor	Reflective and regulatory processes	Goal setting (1.1)*, self‐monitoring (2.3)*, behavioral practice/rehearsal (8.1)*,graded tasks (8.7)*, restructuring the physical environment (12.1)*; instruction on how to perform a behavior (2.1)*
6	Take it outdoors Budget‐friendly family activities	The influence of environment on behavior, focus on outdoor Changing one's environment to promote PA, focus on outdoor	Regulatory processes	Goal setting (1.1)*, self‐monitoring (2.3)*, behavioral practice/rehearsal (8.1)*,graded tasks (8.7)*, restructuring the physical environment (12.1)*; instruction on how to perform a behavior (2.1)*
7	Family self‐care Activity highlight – Geocaching	Building your social support network	Regulatory processes	Goal setting (1.1)*, self‐monitoring (2.3)*, behavioral practice/rehearsal (8.1)*, social support (3.3)*
8	Healthy habits Activity highlight – Hiking	Introducing habit Habit and PA How to form a habit (repetition, scripts, environmental cues)	Reflexive processes	Goal setting (1.1)*, self‐monitoring (2.3)*, behavioral practice/rehearsal (8.1)*
9	Exercise identity Activity highlight – Cycling	Introduce exercise identity Ways to increase exercise identity	Reflexive processes	Goal setting (1.1)*, self‐monitoring (2.3)*, behavioral practice/rehearsal (8.1)*,graded tasks (8.7)*, social support (3.3)*, valued self‐identity (13.4)*, habit formation (8.3)*
10	Leading by example Our last dance	Self‐monitoring tools Action and coping planning Goal setting The importance of enjoying PA	Reflective, regulatory, and reflexive processes	Goal setting (1.1)*, self‐monitoring (2.3)*, behavioral practice/rehearsal (8.1)*,graded tasks (8.7)*, social support (3.3)*, valued self‐identity (13.4)*
JITAI	Making physical activity pleasant Improve YOUR ability to support your child's physical activity Improving opportunities for physical activity support	Strategies to make PA enjoyable Highlight positive experiences to build motivation	Reflective processes	Information about emotional consequences (5.6)*, focus on past success (15.3)*, behavioral practice/rehearsal (8.1)*
JITAI	Active living: Strategies for parents to promote physical activity Plan ahead for success What to do when life happens Support from our surroundings Using Fitbit to its full potential	Strategies for planning daily PA Overcoming barriers Using social and physical environments to support PA	Regulatory processes	Action planning (1.4)*, problem solving (1.2)*, self‐monitoring (2.3)*, restructuring the physical environment (12.1)*
JITAI	Well done! Support superstar!	Celebrate successes and reinforce progress Strategies for sustaining motivation and building long‐term habits	Reflexive processes	Positive reinforcement (10.4)*, habit formation (8.3)*, valued self‐identity (13.4)*

*Note*: The traditional modules are organized by the week they appear. The three rows of JITAI modules were available throughout the intervention based on survey responses.

Abbreviations: M‐PAC = Multi‐Process Action Control; BCT = behavior change technique; PA = physical activity; FITT = Frequency, Intensity, Time, Type; JITAI = just‐in‐time adaptive intervention *The numbers in brackets indicate the behavior change technique for reporting in behavior change interventions (Michie et al., 2013).

### Phase 2: intervention development (December 2022–March 2023)

#### Step 4: ideate implementation strategies

We co‐developed implementation strategies for the app adaptive content and features (e.g., rewarding systems, activity goal suggestions) using the Pathverse platform. We focused on several core areas to optimize the app's design and functionality. First, we ensured that the content aligned with the users' needs within the M‐PAC framework by initially focusing on reflective and regulatory processes, followed by a gradual progression toward reflexive processes. To support this layered approach, content addressing each of these processes was integrated throughout the weeks in a way that built upon one another. This ensured consistent engagement, while also introducing tailored modules based on JITAI algorithms that adapted to each user's responses. These personalized modules provided specific guidance depending on the type of support the parents required concerning the M‐PAC framework, encouraging sustained parental support for their child's PA. Second, we selected app colors, graphics, and layout elements based on team and end‐user feedback to ensure an appealing and easy‐to‐use user interface. Third, in alignment with the M‐PAC framework, through feedback from families, we incorporated a variety of family‐based PA challenges and rewards. These challenges were designed to be enjoyable, attainable, and at varying intensities, with families able to select new goals every week. This flexibility supported the development of intention by allowing families to tailor activities to their interests and capabilities. To strengthen regulatory processes, gamification elements were introduced, enabling families to earn points for completing weekly modules and family challenges. This point‐based system was intended to reinforce positive behaviors and encourage sustained engagement with the app, promoting action control through rewards. Additionally, we added social features through a chat forum to foster community building and peer support.

#### Step 5: prototype potential products

**FIGURE 2:**
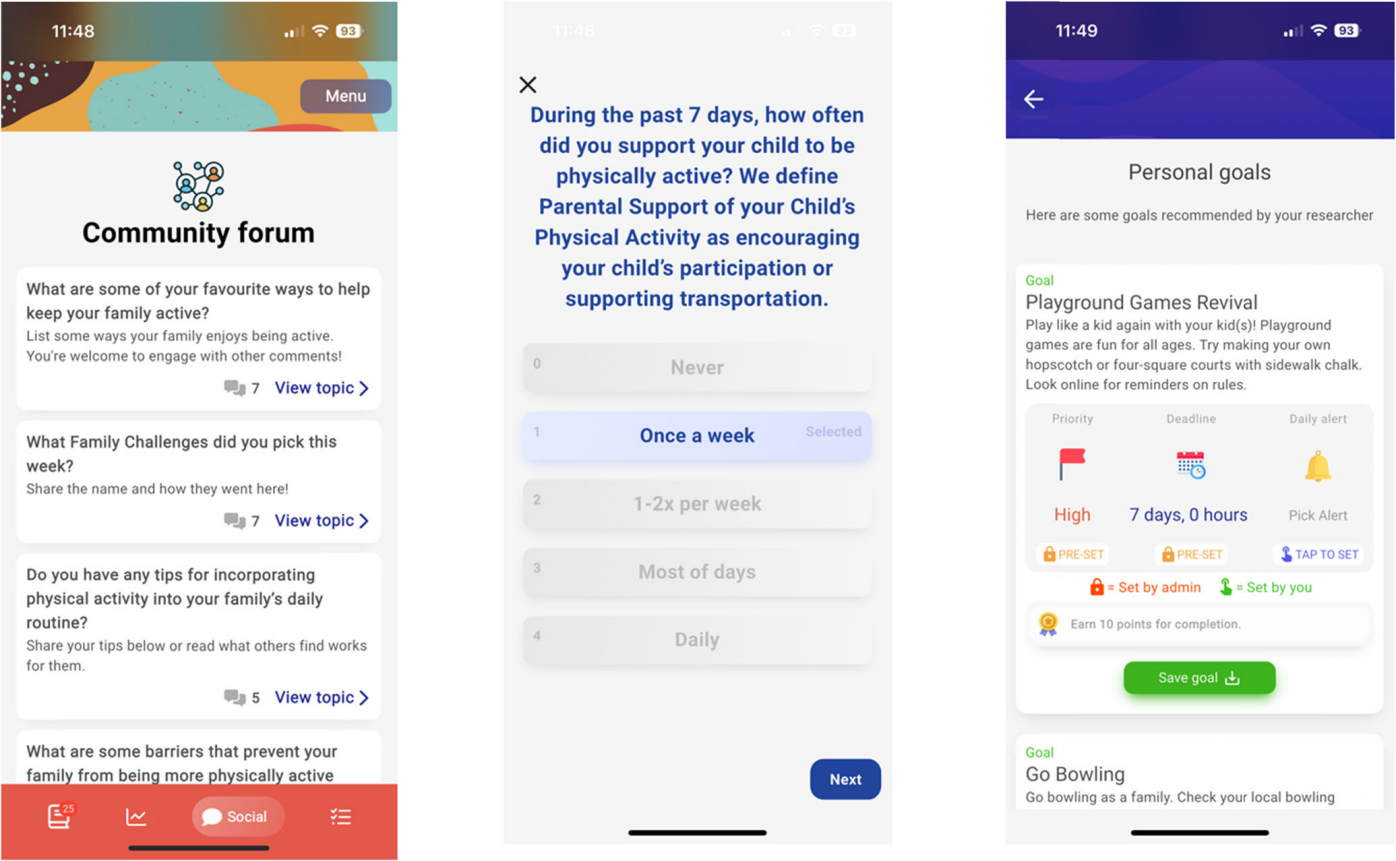
Prototype of the app before usability testing.

The Movement for Active Families app, built using the Pathverse platform, incorporated all key features identified in earlier planning stages (Figure 2). These included: (1) tailored weekly content to improve parental support for child PA, (2) engaging weekly family challenges, (3) reward points for completing lessons and activities, (4) a forum for discussions, (5) a self‐monitoring tool for PA tracking, and (6) notifications and reminders for weekly modules. App development spanned four months (December 2022–March 2023) and took an estimated 320 hours (See Table 5). Specifically, 240 hours were dedicated to creating the JITAI app using the Pathverse platform, and 80 hours were spent on quality checks and fixing the app based on user feedback.

**TABLE 5:** Overview of the app development process and resource requirement.

Timeline	Overview of tasks	Hours
Dec 2022	Upload content for 20 M‐PAC lessons and 30 family challenges. Customize app theme and graphics	50
Dec 2022‐Jan 2023	Internal testing of content	30
Jan 2023	Make changes based on feedback (amount of content per page, grammar/spelling, images/icons used throughout, bug fixes)	20
Jan‐Feb 2023	Create decision trees for dynamically tailored content. 13 lessons were created	50
Feb 2023	Upload dynamic content into Pathverse	70
Feb‐March 2023	Assign testers for each possible decision tree route (nine options) and perform internal testing	50
March 2023	Make changes based on feedback	30
March 2023	Implement gamification into program (points for completing lessons, family challenges)	20

*Note*: JITAI = just‐in‐time adaptive intervention; M‐PAC = Multi‐Process Action Control.

The app development process began with uploading generated content for 20 M‐PAC modules and 30 family challenges onto the app, along with customizing an app theme and graphics. The content was organized to ensure consistency throughout the app, and a cohesive set of images and app icons was purchased from FlatIcon (Freepik Company S.L.) to ensure consistent imagery throughout the program. While uploading the content, the research assistant (AW) rapidly tested to ensure the content uploaded was optimized for viewing on smartphones (e.g., no text cut‐off) before advancing to internal team testing. During the internal testing, our team reviewed module content and app functionalities and usability and reported any errors or bugs to AW and the development team. After these changes were made to the app, AW set the app logic for the JITAI decision trees created in the previous step. This process involved creating a decision tree algorithm involving 13 unique paths based on user choices. Each decision path was then mapped, uploaded, and tested to ensure logical flow and proper sequencing. Upon completing the internal team testing, we made final content adjustments and implemented the gamification elements mentioned by families during phase 1. A member of our research team then implemented points into the app to be earned when participants completed modules and family challenges. Families were able to redeem points for prizes (e.g., water bottle, jump rope) at the end of the intervention.

## DISCUSSION

### Main findings

This study aimed to describe the co‐design and development of the Movement for Active Families JITAI app and discuss the resources required for this process. The application of the IDEAS framework facilitated an iterative and efficient co‐design approach, ensuring that parent input was central to the app's development. Our findings emphasize the importance of involving parents in the co‐design process, as their insights were instrumental in shaping the content and guiding the selection of appropriate behavior change techniques to incorporate. Overall, the combination of the IDEAS and the M‐PAC frameworks, along with the no‐code development tool, was effective in creating the Movement for Active Families JITAI app.

Three key factors contributed to the overall success of the Movement for Active Families JITAI development. First, the co‐design process with target users was instrumental in identifying and incorporating essential features, such as a variety of multimedia content and an intuitive, easy‐to‐navigate interface. Using the IDEAS framework for this process facilitated an efficient and structured approach to integrating user feedback. The framework helped streamline the co‐design process by emphasizing user empathy, behavior change theories, and iterative feedback. The features identified by families during semi‐structured interviews were consistent with previous family‐based PA mHealth apps, including activity trackers, gamification elements (Schoeppe et al., 2023; Wunsch et al., 2020), and diverse health content delivered through various multimedia formats (Karssen et al., 2021). Interventions incorporating components such as PA trackers and incentives for achieving PA goals have been associated with high retention rates (95%) in family‐based interventions targeting moderate‐to‐vigorous PA (Schoeppe et al., 2020) and were therefore considered when designing this study.

Second, this study demonstrated the flexibility of the M‐PAC framework, which was used to tailor the app's JITAI content. In previous mHealth and web‐based studies (Hartson et al., 2022; Hartson et al., 2024; Liu, Husband, et al., 2019; Willms et al., 2023a), content using the M‐PAC framework has typically been delivered in a linear sequence (e.g., targeting reflective processes such as intention in the initial weeks, followed by regulatory and reflexive processes toward the end of the intervention). By adopting the M‐PAC framework into a JITAI, we were able to dynamically personalize the delivery of both theoretical constructs and BCTs (e.g., goal setting, self‐monitoring, social support) to meet the unique needs of each family. In this way, the intervention was designed to be tailored not only to the timing but also to the M‐PAC construct and the associated BCT(s) that the family needed that week (e.g., intention formation, action control, habit development). Further, these BCTs are among the most frequently applied in PA JITAIs; however, prior interventions have often used them without explicit theoretical grounding or consistent personalization logic (Cotie et al., 2025). To our knowledge, this is the first published study to dynamically implement the M‐PAC framework within the co‐design of a JITAI. In future family‐based intervention studies, the M‐PAC framework could serve as a foundation for developing adaptive interventions that respond to shifts in motivation and behavior over time. The M‐PAC framework provides a theoretical basis for operationalizing BCTs linked to regulatory processes (e.g., goal setting, self‐monitoring, problem solving, social support) as well as those that may facilitate reflexive processes (e.g., habit formation, behavioral practice, identity associated with changed behavior) (Rhodes, 2017).

Third, we were able to rapidly prototype the JITAI using the Pathverse platform in just 320 hours. This is consistent with the opinion of expert developers that low‐code and no‐code platforms lead to faster, easier development with lower costs (Luo et al., 2021). However, whereas commercial low‐code platforms, such as Microsoft Power Platform, can involve expensive licensing fees (Luo et al., 2021), using the Pathverse research platform circumvented these costs. This adds to the growing evidence that low‐code and no‐code development tools can facilitate artificial intelligence‐based analysis in social science research (Sufi, 2023) by enabling domain experts and “citizen developers” to leverage dynamic, real‐time decision‐making based on user data. Currently, there are limited “no‐code” platforms available; however, as this technology advances, it will significantly improve JITAI development efficiency (Willms et al., 2024). The next steps include exploring ways to integrate emerging advancements in no‐code technology, such as the global positioning system and/or data from wearable sensors, into the Pathverse platform and conducting usability testing to ensure seamless adoption by researchers.

### Limitations

The small sample size of parents involved in the co‐design process may limit the generalizability of our findings. We encountered challenges in recruiting and scheduling families for focus group discussions. Thus, we transitioned to semi‐structured interviews with families to meet their scheduling needs, as it was more feasible to accommodate each family's availability. While the diverse cultural backgrounds of participants provided valuable insights, future studies with larger and more representative samples are needed to ensure the app meets the needs of a broader audience. This study also marked the first use case of JITAI within the Pathverse platform, which introduced some technical limitations. For example, slow loading times were encountered when creating and duplicating modules, which impacted the development process. These issues were reported to the Pathverse team, who are now working on platform improvements for future JITAI implementations.

## CONCLUSION

This study described the co‐design development process of the Movement for Active Families JITAI app. Our findings highlight the importance of engaging participants in the co‐design process to gather essential features tailored to the end‐users' needs. This is also one of the first studies to implement the M‐PAC framework in a dynamic JITAI context, providing a promising foundation for applying M‐PAC to other JITAI interventions. The use of the Pathverse no‐code platform enabled our team to rapidly prototype the app and significantly reduced the development time typically required for a complex mobile health intervention like a JITAI. As no‐code technologies continue to advance, they hold great promise for further improving the efficiency and accessibility of JITAI app development. This study lays the groundwork for future research to evaluate the efficacy of the Movement for Active Families JITAI app.

## CONFLICT OF INTEREST STATEMENT

Dr. Sam Liu declares that he is a co‐founder of Pathverse Inc., but did not receive any funding from Pathverse to conduct this study.

## ETHICS STATEMENT

Ethical approval was obtained from the University of Victoria Research Ethics Board (protocol number 22–107). Written informed consent was obtained from all participants before participation.

## PERMISSION TO REPRODUCE MATERIAL FROM OTHER SOURCES

This study was not formally registered. The analysis plan was not formally pre‐registered. De‐identified data from this study are not available in a public archive. De‐identified data from this study can be made available (as allowable according to institutional IRB standards) by emailing the corresponding author. There is no analytic code associated with this study. Materials used to conduct the study are not publicly available.

## CLINICAL TRIAL REGISTRATION

This phase of the study was not registered.

## Data Availability

The data that support the findings of this study are available upon reasonable request from the corresponding author. Restrictions may apply to data availability due to privacy or ethical concerns.
